# Toward Mental Effort Measurement Using Electrodermal Activity Features

**DOI:** 10.3390/s22197363

**Published:** 2022-09-28

**Authors:** William Romine, Noah Schroeder, Tanvi Banerjee, Josephine Graft

**Affiliations:** 1 Department of Biological Sciences, Wright State University, Dayton, OH 45435, USA; 2 Department of Leadership Studies in Education and Organizations, Wright State University, Dayton, OH 45435, USA; 3 Department of Computer Science, Wright State University, Dayton, OH 45435, USA

**Keywords:** electrodermal activity, galvanic skin response, cognitive load, mental effort, wearable sensor

## Abstract

The ability to monitor mental effort during a task using a wearable sensor may improve productivity for both work and study. The use of the electrodermal activity (EDA) signal for tracking mental effort is an emerging area of research. Through analysis of over 92 h of data collected with the Empatica E4 on a single participant across 91 different activities, we report on the efficacy of using EDA features getting at signal intensity, signal dispersion, and peak intensity for prediction of the participant’s self-reported mental effort. We implemented the logistic regression algorithm as an interpretable machine learning approach and found that features related to signal intensity and peak intensity were most useful for the prediction of whether the participant was in a self-reported high mental effort state; increased signal and peak intensity were indicative of high mental effort. When cross-validated by activity moderate predictive efficacy was achieved (AUC = 0.63, F1 = 0.63, precision = 0.64, recall = 0.63) which was significantly stronger than using the model bias alone. Predicting mental effort using physiological data is a complex problem, and our findings add to research from other contexts showing that EDA may be a promising physiological indicator to use for sensor-based self-monitoring of mental effort throughout the day. Integration of other physiological features related to heart rate, respiration, and circulation may be necessary to obtain more accurate predictions.

## 1. Introduction

One’s ability to self-regulate their own learning may be pivotal to their long-term academic and professional success, as well as for meeting their own informal learning goals. It is not surprising then that the study of self-regulated learning has been on-going for more than two decades [1,2]. Researchers have conducted a variety of studies around self-regulation in an effort to better understand the construct, and more recently researchers have been investigating its intersection with technology [3].

One rapidly evolving technology is wearable sensors. These have transformed from relatively invasive devices used in healthcare or research settings to now being an integral part of popular consumer devices, such as smartwatches. In the past few years, researchers have begun synthesizing the literature around the use of wearable sensors in educational settings, with a focus on the impacts on learning [4] and their use in learning analytics [5].

As an interdisciplinary team of educational researchers and computer scientists, we are focused on the intersection of self-regulation, learning analytics, and wearable sensors, with the end goal of being able to develop a system that helps students regulate their own learning process based on feedback from a wrist-worn device. To date, research around the use of wearable sensors for predicting learning-relevant outcomes has produced promising results. For example, researchers have found success using multi-modal physiological measures to differentiate between different activities and predict mental focus [6], as well as predicting perceived learning [7].

While research around the use of wearable sensor data in relation to helping students self-regulate their learning is promising, it is also clearly in its infancy as there are many research problems to be solved. For example, it is not clear what type of physiological data are most helpful for predicting any particular outcome, regardless of whether it is a cognitive outcome (like learning) or a perceptive outcome (like perceived mental effort). Furthermore, there are also ongoing questions as to how to process physiological data from wearable sensors, which types of data are most appropriate for predicting different outcomes, and even what algorithms should be used to accurately classify and predict different types of outcomes.

Given these outstanding broad and persisting questions, in this study we take a deep, exploratory dive into one particular type of data, electrodermal activity (EDA), and its relationship with perceived mental effort, a well-established educational construct associated with cognitive load theory [8,9]. Specifically, in this study we examine what types of EDA features are most useful for predicting perceived cognitive load.

## 2. Literature Review

### 2.1. Cognitive Load Theory

Prominent in the educational literature is cognitive load theory, which describes how we allocate working memory resources to different cognitive tasks. Specifically, cognitive load can be expended on tasks that are intrinsic to the learning task (i.e., intrinsic cognitive load), including the construction of mental models (i.e., germane cognitive load), or cognitive load can be expended on processing design elements that are not necessary for learning the material, but rather are present due to less than optimal instructional design decisions (i.e., extraneous cognitive load) [8,9,10].

Studies centered on cognitive load theory can take many forms. A study could involve manipulating an individual design variable that is expected to influence either extraneous load or intrinsic load, and then examining learning outcomes to infer causality. Alternatively, in some studies researchers manipulate similar variables but measure cognitive load through self-reports [11], secondary tasks [12], or physiological measures [13].

Despite the prominence of cognitive load theory in the literature, it is not without critique, and measuring cognitive load in a psychometrically sound way may not be a straightforward task. One such critique is that measuring self-reported cognitive load is quite challenging, and differentiating the three types of cognitive load via self-report may not be possible [14]. That said, there are scales that have some validity evidence that differentiate the types of load, but some questions still exist as to if they are measuring the construct or indicators of the construct [15]. In addition, there are other self-report measures for constructs which are proxy to cognitive load, such as mental effort. Measures of mental effort often act as an indicator of overall cognitive load [16], as opposed to the individual types of cognitive load. One concern with these types of measurements, outside of not being able to differentiate the types of cognitive load, is the question of what is considered too much cognitive load [17].

Since there is currently no ground truth for cognitive load outside of self-reports, they are still a very common, if not the most common, measurement of cognitive load. This is despite the fact that the learner must either interrupt their task to complete the self-report or complete the self-report after the task is completed. However, these issues surrounding self-reported cognitive load have, in part, caused researchers to pursue other avenues of cognitive load measurement, such as secondary tasks or physiological measures. These efforts are noteworthy, but there are situations in which completing secondary tasks may distract from the learning task [18], and there is still a limited body of research around physiological measures that can be measured non-intrusively. Recently, however, researchers have found success using wrist-worn sensors to measure physiological signals to predict mental effort [19]. Further, researchers recently argued that EDA may be a strong physiological measure to use as an indicator of cognitive load [20]. In the next section, we examine how EDA has been used to measure cognitive load and conceptually related measures of mental effort and mental workload.

### 2.2. The Use of EDA to Measure Mental Effort

While the term mental effort is often associated with cognitive load theory, in other areas of the literature it is common to encounter the term mental workload. These terms describe similar constructs [6] and in fields outside of education, mental workload is often measured through physiological means [21] since the autonomic nervous system responds to changes in mental workload, and these changes happen without the individual knowing the changes are occurring [22]. In relation to grain size of measurement, it is important to note that these measures are viewed as an index of workload [23], and perhaps should not be viewed as the exact amount of workload or mental effort.

That said, research in the area is relatively scarce. A recent systematic review found only seven studies that used measures related to the skin in regard to studies of mental workload [21]. Some studies in this area, however, have shown promising results. For example, one study found that galvanic skin response (GSR) could indicate differences between different levels of cognitive load in a traffic control management task [24], and a different study found that GSR could predict mental workload changes with 75% accuracy [25]. While few studies have investigated education-related constructs directly, some individual studies have found promising results. For example, one study used math and reading tasks and found that GSR differentiated between different task difficulties when scores were normalized across participants [26], a further study found similar results [27], and another noted that “EDA seems to be related to mental effort [28] (p. 57).”

### 2.3. The Present Study

Existing research has shown a promising connection between EDA and the prediction of cognitive load-related outcomes such as mental effort or mental workload. However, these studies have used a variety of types of data and algorithms to analyze their data. In fact, researchers have argued that there was not yet a specific machine learning algorithm that has consistently performed well to analyze physiological data in relation to mental effort [19]. Agarwal and colleagues [19] highlighted the efficacy of deep-learning methods as an approach to analyzing raw measures in near real-time; however, we further argue that the question of which algorithm to use may be premature, as there is not consensus on which types of data are most useful. At this stage, what is needed are in-depth, exploratory studies examining different features of different types of physiological data in order to understand which are most useful for prediction. Based on prior work that has shown promising results with EDA and predicting cognitive load-related outcomes, and given cognitive load theory’s long history and prominence in the literature, in this study we explore different types of EDA features in relation to measures of mental effort to build an understanding of what types of EDA features are most useful for predicting mental effort.

## 3. Methods

### 3.1. Experimental Design and Data Collection

Toward better understanding how the EDA signal can be used to predict mental effort, and the efficacy of the signal in generalizing predictions across different activities within an individual, we utilized a dataset on a single participant from previous work [19] which employed a single-participant study design [29]. Longitudinal single-participant study designs have several advantages which make them desirable for understanding how sensors perform in the real world outside of the laboratory. First, focusing on a particular case facilitates context-specific generalizability which allows for more direct application to similar cases than a traditional cross-sectional design which focuses on collecting limited information across a large number of participants [30]. Toward our goal of understanding the efficacy of EDA for predicting mental effort over a semester in a real-world educational setting, it makes sense to collect in-depth data from a single participant who is engaged with high fidelity in the college experience. In sum, our purpose is not to generalize across different types of participants, but rather to understand in detail how the sensor works over an extended time frame for a single participant which will facilitate informed hypotheses in research on participants with similar profiles [30]. Case study methods have been applied in research on activity monitoring with non-wearable sensors [31] but more work using these methods is needed to better understand how wearable sensors work to help us measure mental processes in real life.

The participant in this study was a 19-year-old Caucasian female college student, and data were collected in the context of coursework and daily activities related to the pursuit of a Medical Lab Sciences degree at a research-intensive university in the Midwestern United States. Specifically, data were collected in the context of 91 activities related to the four courses she was taking during the Fall 2020 semester, Cell Biology, Music, Genetics, and Organic Chemistry, and activities she undertook outside of the classroom, which we termed “Everyday Activities”. These ranged from leisure activities such as scrolling social media and watching television to activities of daily living such as cooking and eating, to more cognitively intensive activities such as tutoring and reading. The data collection period lasted over 3 months (from 1 September 2020 to 3 December 2020) during the course of the Fall 2020 academic semester. The activities ranged in duration between 804 and 7869 s, with an average of 3651 s (SD = 1496 s). Across the 91 activities, a total of 332,246 s of EDA data were logged at a 1 Hz sampling rate.

**Figure 1:**
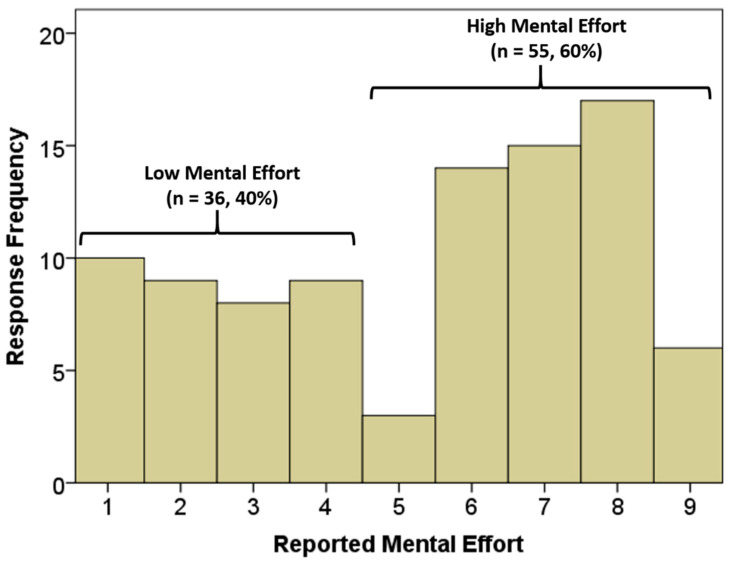
Histogram of mental effort measures for the 91 activities based on Paas’ mental effort scale [16].

The Empatica E4 was used to obtain the EDA data during each activity. The E4 wristband was turned on at the start of each activity and was kept on throughout the entire duration of the activity. Visual inspection of the data indicated false EDA readings attributed to stabilization of the device could be removed by deleting the first 10 s of data from each activity. The participant’s self-reported mental effort rating after each activity was based on Paas’ mental effort 1–9 ordinal scale [16], with 1 being very very low mental effort and 9 being very very high mental effort which we aimed to bin into “high” and “low” mental effort. We binned the data into “high” and “low” based on a histogram of reports which shows a clear break in the participant’s reports between high and low mental effort (Figure 1).

Based on the histogram, we found bimodality in the distribution between regions of low and high mental effort. Based on this distribution, we considered ratings of 1–4 as indicative of “low mental effort” and ratings of 5–9 as indicative of “high mental effort” for subsequent analyses.

### 3.2. Extraction and Explanation of the EDA Features

The EDA features were extracted from the 91 activities using Stata 13 and the MATLAB package Ledalab which is available online (http://www.ledalab.de, accessed on 16 July 2022). Ledalab was used for continuous decomposition analysis (CDA), which is a method for extracting phasic information from the underlying EDA signal across the 91 activities [32].

The skin response can be considered as the sum of two components: the phasic component (also called the Skin Conductance Response $s_{p}\left(t\right)$), as well as the tonic component (also called the Skin Conductance Level $s_{t}\left(t\right)$). The phasic component further comprises the standard linear convolution of the sudomotor SNS innervation ($d_{p}\left(t\right)$) and the response triggered by the driver ($r\left(t\right)$) [33].

$$s\left(t\right)=s_{p}\left(t\right)+s_{t}\left(t\right)=d_{p}\left(t\right)*r\left(t\right)+s_{t}\left(t\right)$$

This level of decomposition allows us to explore the Skin Conductance Response (SCR) and Skin Conductance Level (SCL) features independently, which allows us to extract components that are less likely to be dependent on other factors such as skin temperature [34]. This method of decomposition yielded two CDA-specific features: amplitude sum and skin conductance response, which are measures of peak intensity, and two global features, EDA global mean and global max deflection, which are measures of signal and peak intensity, respectively (Table 1).

We calculated additional features using Stata 13 based on the z-standardized tonic EDA signal. In order to convert raw measures to z-standardized measures, we calculated the participant’s grand mean and standard deviation across all 91 activities and standardized each EDA measure with respect to those values [35]. Within each activity, we calculated the mean and median z-score as measures of signal intensity. In addition, we calculated the standard deviation, interquartile range, and coefficient of variation as measures of relative signal dispersion within each activity. Skewness, kurtosis, and 99th percentile values were used as additional measures of peak intensity within each activity. In sum, we ended up with three measures of signal intensity (EDA z-score mean and median, EDA global mean), three measures of signal dispersion (standard deviation, interquartile range, and coefficient of variation), and six measures of peak intensity (skewness, kurtosis, 99th percentile, amplitude sum, global max deflection, and skin conductance response), for a total of twelve features which were evaluated for prediction of mental effort.

### 3.3. Evaluation of Importance and Significance of Features

Given our interest in interpretable machine learning, we utilized the logistic regression model within both inferential and machine learning frameworks. The first step in this process was to use the features in Table 1 to build a model for discrimination between high and low mental effort which yielded predictions that are significantly stronger than simply using the most commonly reported mental effort state (mode = high mental effort, see Figure 1). In machine learning applications, this is sometimes referred to as model bias. The 95% confidence level was used to test the extent to which the model performed better than using the model bias alone outside of chance. Assuming such a model could be constructed, we then proceeded to evaluate classification performance using the logistic regression model. Since the interest was in the efficacy of EDA features calculated from past activities to predict future activities, we used activity-fold cross-validation as a training-testing framework. With this procedure, one activity was excluded from the data for testing and the model trained on data from the other activities was tested on data from the hold-out activity. This was repeated across all 91 activities. Area under the receiver operator characteristic (ROC) curve (AUC), precision, recall, and F1 score were used as metrics of classifier performance.

## 4. Results

### 4.1. Building the Logistic Regression Model

All twelve of the features in Table 1 were entered into the model, which resulted in a non-significant model (χ^2^ (12) = 16.5, *p* = 0.17). Hence, in order to reduce model complexity sufficiently to obtain a model with a significant chi-square value, we needed to remove five non-significant features: standard deviation (*p* = 0.30), 50th percentile (*p* = 0.79), interquartile range (*p* = 0.26), amplitude sum (*p* = 0.83), and coefficient of variation (*p* = 0.75). Removing these had no significant effect on model fit (χ^2^ (5) = 1.72, *p* = 0.89). This left a model with seven features: mean z-score, skewness, kurtosis, 99th percentile, global max deflection, global mean, and SCR. This model predicted mental focus significantly better than the intercept at the 95% confidence level (χ^2^ (7) = 14.8, *p* = 0.039). To further improve prediction using these existing features, we attempted to add 2-way interactions of these variables to the model in a forward stepwise fashion using the likelihood ratio test at the 95% confidence level. Using this procedure, one two-way interaction, skewness by 99th percentile, improved model fit (likelihood ratio χ^2^ (1) = 4.6, *p* = 0.031). After adding this to the model, we obtained a final 8-feature model which predicted focus significantly better than the intercept at the 95% confidence level (χ^2^ (8) = 19.4, *p* = 0.013) (Table 2).

### 4.2. Analysis of Relationships between the EDA Features and Mental Effort

Table 2 indicates that the most important features for discriminating between high and low mental effort address signal intensity and peak intensity. With respect to signal intensity, the mean of the EDA z-scores appears to have a negative relationship with high mental effort (OR = 0.011, χ^2^ (1) = 5.34, *p* = 0.021); on the other hand, the EDA global mean (OR = 3.93 × 10^9^, χ^2^ (1) = 3.76, *p* = 0.053) has a positive effect size which is significant at the 90% confidence level. This dichotomy may be due to the difference in how these are calculated; the former as a difference between the participant’s average level across the activity duration, and the latter as an average level due to EDA responses during the activity [32]. With respect to peak intensity, global max deflection has a strong positive relationship with mental effort (OR = 1.22 × 10^24^, χ^2^ (1) = 5.81, *p* = 0.016) as does the 99th percentile of the EDA z-scores (OR = 8.99, χ^2^ (1) = 5.28, *p* = 0.022). This demonstrates a strong relationship between peak intensity and mental effort. However, we find that skewness tempers this effect; once the 99th percentile value is accounted for, we find that more positive skewness (i.e., a bulk of lower values with strong positive outliers) tends to lower the effect (OR = 0.513, χ^2^ (1) = 3.76, *p* = 0.052 for the interaction and OR = 0.208, χ^2^ (1) = 5.00, *p* = 0.025 for the effect of skewness as the main effect). This interaction between the 99th percentile and skewness is potentially important as it distinguishes between EDA peak and EDA storm activity (which constitutes multiple peaks in rapid sequence). Specifically, a high 99th percentile value and lower skewness indicate a larger number of high values within the distribution; on the other hand, the same 99th percentile with higher skewness indicates phasic activity where most of the measures are still tending toward the lower end of the distribution. Finally, the least important variables appeared to be kurtosis (OR = 1.36, χ^2^ (1) = 2.72, *p* = 0.099 and SCR (OR = 1.93, χ^2^ (1) = 2.10, *p* = 0.15), which both had positive effect sizes. Kurtosis was significant at the 90% confidence level, indicating some tendency for greater density or unimodality of EDA values during high mental effort activities.

### 4.3. Efficacy of the EDA Features for Classification of Mental Effort

When cross-validated by activity, predictive efficacy was moderate (AUC = 0.63, F1 = 0.63, precision = 0.64, recall = 0.63), but still yielded predictions which were significantly stronger than using the model bias alone (AUC = 0, F1 = 0.46, precision = 0.37, recall = 0.60). Analysis of the receiver operator characteristic (ROC) curve shows that a classification cutoff of 0.53 optimizes both sensitivity (0.66) and specificity (0.67). However, toward generating a useful sensor-based prediction for mental effort, it is important to consider whether we want a sensitive model which is able to identify instances high mental effort accurately, or whether we want a more specific model which more accurately identifies instances of low mental effort. A sensitive model could be obtained by adjusting the classification cutoff to 0.44. Such a model would successfully identify 70% of the high mental effort states while correctly identifying 50% of the low mental effort states, meaning that it would tend to generate false alarms. On the other hand, a specific model could be obtained by adjusting the classification cutoff to 0.57. This model would correctly identify 72% of the low mental effort states while correctly identifying 50% of the high mental effort states, meaning that it would miss about half of a user’s high mental effort activities but would generate far fewer false alarms. Toward having a device that is non-distracting and useful in day-to-day activities which is also able to log temporal changes in mental effort over the long term, we argue that a more specific classification framework would be preferred which would minimize false alarms while also successfully logging many instances where high mental effort was achieved.

## 5. Discussion

The use of wearable sensors has been developing rapidly over the past decade. While current devices focus primarily on physical exercise, fitness, and sleep quality, current research has demonstrated that useful predictions for constructs related to cognitive load, such as mental workload and mental effort, are within reach. In this study, we focus on the efficacy of using features derived from a user’s EDA signal for generating predictions of mental effort which generalize across different activities for a single participant over a semester-long time period. This gives an approximation of how a device trained on a single participant’s EDA readings would work for prediction of mental effort for that participant’s activities in the future; but it is likely that this participant’s model would not generalize directly to other users. With this being said, these data corroborate other research [19,24,25,26,27] suggesting that increases in EDA are indicative of higher levels of mental effort. In this sense, these general observations regarding the effect of signal intensity and peak intensity could inform the implementation of an informative prior model within a mental effort tracking device so that the device could be trained and optimized for generating useful predictions with a new user more quickly. In addition, our data show that features related to signal and peak intensity are most efficacious toward generating useful predictions for mental effort which suggests that future research should focus on these.

Although classification efficacy for mental effort using the features derived from this participant’s EDA signal is modest, we argue that a device with modest classification efficacy (i.e., AUC in the 0.6–0.7 range) can be useful if: (1) the classification algorithm is optimized to favor high specificity (i.e., result in fewer false alarms even at the cost of false negatives), and (2) the device is used over a long time period which would elucidate trends over time that could help create causal models that can determine the factors indicating a state of high mental effort in an individual. These two criteria deserve further explanation. We argue that from the perspective of a college student who is regularly engaged in activities requiring high levels of mental effort, a device that more accurately predicts low mental effort activities would be most useful in that it would generate few false alarms while still picking up a portion of the participant’s actual high mental effort activities throughout the day. Although this would limit efforts to provide an accurate single-day estimate of time on the task at high mental effort, it would nonetheless be quite informative for understanding trends in mental effort over an extended period of time provided that the sensitivity and specificity of the device stay constant over time. For example, if a student wanted to track their mental effort during the week of their final exams, the device would likely underestimate the actual number of high mental effort activities; however, it should be able to detect an increase in the number of high mental effort activities relative to previous weeks. This would also be useful in a classroom setting if the instructors were informed if a student achieved high mental effort, with the higher specificity resulting in more conservative but trustworthy information. We also argue that, as mentioned earlier, the individual nature of the mental effort patterns indicates that even with a smaller sample size, we can determine high mental effort in the participants and possibly increasing the sample size need not improve the accuracy of the models.

Toward generating a realistic picture of how EDA is likely to perform for detection of mental effort in a real-world setting, a strength of this study design is that it focused on an uncontrolled setting over a significant time duration. It is well-known that many things can affect a person’s EDA readings that are unrelated to mental efforts such as stress or changes in mental health [36,37], medication use, caffeine intake, and changes in temperature and humidity [38]. The extended study of a participant in their natural environment allows these circumstances to be factored into the classification error; in this sense, we expect larger errors in a study like this than we would expect in educational studies focusing on a research lab setting, or even a specific classroom setting. Within our between-activity cross-validation procedure, our reports of classifier performance have strong internal validity for this participant, and we argue that these are likely to be representative of other participants with similar backgrounds and goals.

In summary, the EDA features used in this study may be useful for predicting relative change, but stronger classification is needed before we are able to make accurate predictions for actual time on task. To the end of further improving prediction, one approach may be to try additional types of EDA features. In this study, we focus on features from the tonic and phasic signals resulting from the CDA approach. However, other decomposition approaches such as Discrete Decomposition Analysis (DDA) will yield different features. There is currently little agreement as to the best EDA features to utilize, making use of the EDA signal as much an art as a science at this point in time. However, findings from multiple studies [19,24,25,26,27] have generated a consensus that engagement in tasks requiring higher mental effort can be detected through increases in both the tonic and phasic parts of the signal. It makes sense, then, that although the specific features used may vary by study, those which get at signal and peak intensity will likely provide useful information regarding a user’s mental effort. It also makes sense that the more unique features we can use to get at signal and peak intensity, the better the resulting predictions will be. Another potential avenue to explore is the use of a higher-frequency EDA signal. Since EDA is a slow signal (i.e., changes in the phasic component of the EDA signal occur on the order of seconds and changes in the tonic component occur on the order of minutes) and the activities evaluated with this participant were over an hour in duration on average, a 1 Hz frequency signal provided a sufficient number of measures during each activity to provide reliable estimates of the distributional features of user’s skin conductance level and skin conductance responses during the activity. However, a higher frequency EDA signal, such as 50–100 Hz, may be needed to detect minute-by-minute transitions in mental effort.

Future experimental work on the efficacy of using the EDA signal for the prediction of mental effort can be discussed in light of the limitations of our current study design. A single-user context helps us understand how a mental effort sensor can be trained on a single participant and gives insight into the EDA features which may be most useful for mental effort classification in a longitudinal context [30]. Although we expect the findings from this case to generalize to other university students in terms of the types of EDA features that are most useful for mental effort classification and the classifier performance that might be obtained over a single-semester training duration, we would expect this participant’s model to perform poorly on a new participant given that both tonic EDA and galvanic skin response is highly variable from one participant to the next due to physiological diversity and the range of unique environmental factors to which different individuals are exposed [35]. Toward gaining a stronger understanding of individual differences, a useful next step would be to extend a study like this to a multiple case study design which focuses on similarities and differences in the models among different cases [30]. Through the process of purposeful participant selection, students engaged in different programs of study, which bring with them different types of learning environments and activities, could be compared. Comparison between a small number of cases could be accomplished through descriptive comparisons of the directionality and magnitude of the EDA features with mental effort and the strength of the classifiers using a between-activity cross-validation framework similar to this study.

A larger-scale multiple case study with many participants could support the use of longitudinal mixed effects models where baseline values and trends are treated as between-participant random effects which would enable the inspection of the random effects covariance matrix or use of intraclass correlation coefficients to shed light on the extent of between-participant variability in model intercepts and trends [39]. Whether the researcher chooses to model each participant separately or use a mixed effects approach to modeling the participants together while accounting for individual differences in intercepts and trends, we expect to find the highest variability in the participant baseline or intercept values and comparatively lower (but significant) variability in the magnitude of the relationship between the EDA features and self-reported mental effort. Specifically, we would expect features related to EDA signal and peak intensity to be positively related to mental effort, but also expect that the magnitude of these relationships would vary across different participants. Although the specific models would likely vary from one participant to the next within the above constraints, we see no reason to believe that useful classifications for mental effort could not be achieved for a new participant providing that adequate training data exist to account for that participant’s unique EDA responses. Our data indicate the efficacy of using 91 activities over the course of a semester, but future studies could experiment with shorter or more extensive durations. In any case, we recommend that future studies retain the naturalistic focus of the current study; in other words, concentrating on uses within a student’s natural environment [40] instead of using controlled laboratory settings which will tend to overestimate the performance we are likely to see in the real world.

## 6. Conclusions

In this study, we demonstrate the usefulness of EDA features related to signal and peak intensity for generating predictions for low vs. high mental effort that generalize across 91 activities undertaken by a university student pursuing undergraduate coursework over the course of a semester. We argue that the features used in this study can provide a useful framework for a wearable device to generate inferences for change in mental effort over time provided that the modeling framework prioritizes specificity. However, stronger classification performance would be necessary before such a device could provide accurate estimates for the actual time at high mental effort on a day-to-day basis. We further argue that the model presented in this study could be useful as a prior model (i.e., one that is built favoring a particular set of models a priori as opposed to building a model with an assumption of a uniform prior distribution of possible models) which would expedite the training (although still require it) of such a device on a new participant given multiple studies that have found a positive relationship between mental effort and signal and peak intensity. With this being said, both skin conductance level and skin conductance responses can be highly variable from one individual to another, and so there is likely to be a relatively modest upper limit on the extent to which we are able to generalize predictions across individuals using the EDA signal exclusively. Given that devices such as the Empatica E4 provide other types of physiological data such as heart rate and skin temperature which have been shown to be related to mental effort [6,19], and that there are devices such as the BioPac sensor which, while more invasive than a wrist-worn wearable, would give access to the higher-frequency EDA data that would be needed to predict higher-frequency transitions in mental effort, the current findings may provide a promising cornerstone for future research and development in this area.

## Figures and Tables

**Table 1:** Features extracted from the EDA signal.

Feature	Type	Explanation
EDA z-score mean	Signal intensity	The mean of EDA z-scores for the activity. Indicative of the average EDA response relative to the average across all activities.
EDA Global Mean	Signal intensity	Mean skin conductance value of the EDA response within the activity.
EDA Median	Signal intensity	The median (50th percentile) of the EDA z-scores for the activity. A non-parametric measure of the average EDA response relative to the grand average across all activities.
EDA z-score standard deviation	Signal dispersion	The standard deviation of EDA z-scores for the activity. A measure of the average spread of EDA measures around the mean.
EDA z-score interquartile range	Signal dispersion	The interquartile range of the EDA measures. Higher values indicate more spread toward the center of the distribution.
EDA z-score coefficient of variation	Signal dispersion	The coefficient of variation of the z-scores for the activity. Calculated as the standard deviation divided by the mean, a higher measure implies more dispersion of the EDA signal around the mean.
EDA kurtosis	Peak intensity	The kurtosis of the EDA distribution for the activity. More positive values indicate thinner tails of the distribution, which indicate greater unimodality.
EDA skewness	Peak intensity	The skewness of the EDA distribution for the activity. More positive values indicate outlying data in the positive direction, which is indicative of peak activity.
EDA z-score 99th percentile	Peak intensity	The 99th percentile of the EDA measures for the activity. Higher values indicate higher EDA peak responses.
EDA Amplitude Sum	Peak intensity	Sum of galvanic skin response (GSR) amplitudes for significant GSR’s which are reconvolved from corresponding peaks.
EDA Global Max Deflection	Peak intensity	Maximum positive deflection/impulse of EDA response within the activity.
EDA Skin Conductance Response	Peak intensity	Average phasic driver within the activity. The phasic driver component exhibits a zero baseline and distinct peaks.

**Table 2:** Parameter estimates, odds ratios (OR) and corresponding hypothesis tests in order of variable importance. *p*-values are calculated with respect to H_0_: B = 0, OR = 1.

Feature	B	SE	Wald χ^2^ (df = 1)	*p*-Value	OR
EDAGlobalMaxDeflection	55.46	23.01	5.81	0.016	1.22 × 10^24^
EDAzMean	−4.50	1.95	5.34	0.021	0.011
EDAzp99	2.20	0.96	5.28	0.022	8.99
EDAskewness	−1.57	0.70	5.00	0.025	0.208
EDAskewness × EDAzp99	−0.67	0.34	3.76	0.052	0.513
EDAGlobalMean	22.09	11.40	3.76	0.053	3.93 × 10^9^
EDAkurtosis	0.31	0.19	2.72	0.099	1.36
EDASCR	0.65	0.45	2.10	0.148	1.93
Intercept	−9.96	4.15	5.75	0.016	

## Data Availability

Data are available upon request by contacting the corresponding author. Data are not publicly available due to privacy restrictions.
